# Evaluating possible ‘next day’ impairment in insomnia patients administered an oral medicinal cannabis product by night: a pilot randomized controlled trial

**DOI:** 10.1007/s00213-024-06595-9

**Published:** 2024-05-17

**Authors:** Anastasia Suraev, Danielle McCartney, Nathaniel S. Marshall, Christopher Irwin, Ryan Vandrey, Ronald R. Grunstein, Angela L. D’Rozario, Christopher Gordon, Delwyn Bartlett, Camilla M. Hoyos, Iain S. McGregor

**Affiliations:** 1 Centre for Sleep and Chronobiology, Woolcock Institute of Medical Research, Macquarie University, Sydney, Australia; 2 Lambert Initiative for Cannabinoid Therapeutics, University of Sydney, Sydney, Australia; 3 Faculty of Science, School of Psychology, University of Sydney, Sydney, Australia; 4 Brain and Mind Centre, University of Sydney, Sydney, Australia; 5 Faculty of Medicine, Health and Human Sciences, Department of Health Science, Macquarie University, Sydney, Australia; 6 Faculty of Medicine, Health and Human Sciences, School of Psychological Sciences, Macquarie University, Sydney, Australia; 7 Faculty of Medicine and Health, University of Sydney, Sydney, Australia; 8 Department of Respiratory and Sleep Medicine, Royal Prince Alfred Hospital, Sydney, Australia; 9 School of Health Sciences and Social Work, Griffith University, Gold Coast, Australia; 10 Menzies Health Institute Queensland, Gold Coast, USA; 11 Department of Psychiatry and Behavioral Sciences, Johns Hopkins University School of Medicine, Baltimore, MD USA

**Keywords:** cannabinoids, cognition, driving performance, sleep disorders, next day function

## Abstract

Cannabis and its major constituents, Δ^9^-tetrahydrocannabinol (THC) and cannabidiol (CBD), are being widely used to treat sleep disturbances. However, THC can cause acute cognitive and psychomotor impairment and there are concerns that driving and workplace safety might be compromised the day after evening use. Here, we examined possible ‘next day’ impairment following evening administration of a typical medicinal cannabis oil in adults with insomnia disorder, compared to matched placebo. This paper describes the secondary outcomes of a larger study investigating the effects of THC/CBD on insomnia disorder. Twenty adults [16 female; mean (SD) age, 46.1 (8.6) y] with physician-diagnosed insomnia who infrequently use cannabis completed two 24 h in-laboratory visits involving acute oral administration of combined 10 mg THC and 200 mg CBD (‘THC/CBD’) or placebo in a randomised, double-blind, crossover trial design. Outcome measures included ‘next day’ (≥9 h post-treatment) performance on cognitive and psychomotor function tasks, simulated driving performance, subjective drug effects, and mood. We found no differences in ‘next day’ performance on 27 out of 28 tests of cognitive and psychomotor function and simulated driving performance relative to placebo. THC/CBD produced a small decrease (-1.4%, *p*=.016, *d*=-0.6) in accuracy on the *Stroop-Colour Task* (easy/congruent) but not the *Stroop-Word Task* (hard/incongruent). THC/CBD also produced a small increase (+8.6, *p*=.042, *d*=0.3) in self-ratings of *Sedated* at 10 h post-treatment, but with no accompanying changes in subjective ratings of *Alert* or *Sleepy* (*p’s*>0.05). In conclusion, we found a lack of notable ‘next day’ impairment to cognitive and psychomotor function and simulated driving performance following evening use of 10 mg oral THC, in combination with 200 mg CBD, in an insomnia population who infrequently use cannabis.

**Supplementary Information:**

The online version contains supplementary material available at 10.1007/s00213-024-06595-9.

## Introduction

The increasing legal use of medical and non-medical cannabis products across many jurisdictions has raised important questions regarding road and workplace safety (Arkell et al. 2021; Cole and Saitz 2020). The main intoxicating component within cannabis, Δ^9^-tetrahydrocannabinol (THC), causes characteristic dose-dependent sensory and perceptual changes, and acute impairment in cognitive and psychomotor performance (Bosker et al. 2012; Preuss et al. 2023; Spindle et al. 2021). This can compromise the performance of safety-sensitive tasks such as operating a vehicle, increasing the risk of error, accident, and injury (Ramaekers 2018; Rogeberg 2019; Rogeberg and Elvik 2016). In contrast, cannabidiol (CBD), does not cause cognitive, psychomotor or driving impairment, even at very high doses (e.g., 1500 mg) (McCartney et al. 2022a, 2022b). The duration of such impairment, or the length of time an individual should wait after consuming cannabis before performing safety-sensitive tasks is a critical issue, particularly for those using a THC-based medication by night to treat a sleep disorder.

A recent meta-regression analysis performed by our group found that most driving-related skills in occasional cannabis users recover within ~5 h of inhaling (e.g., smoking, vaporizing) and ~8 h of orally ingesting 20 mg THC (McCartney et al. 2021). The effects of oral THC take longer to appear and disappear due to its slower rate of absorption. Moreover, there is evidence that CBD can inhibit the metabolism of THC when orally administered, which could increase the magnitude and extend the duration of impairment related to THC (Zamarripa et al. 2023). However, this meta-regression analysis did not include performance tests conducted >12 h after THC use. In a recent systematic review, we showed that very limited evidence exists to support the assertion that THC use impairs ‘next day’ performance (>8 h after THC or cannabis use) (McCartney et al. 2022a, 2022b). We also revealed a lack of rigorous, high-quality studies investigating ‘next day’ effects of THC. Indeed, *none* of the studies were found to have low risk of methodological bias or to have studied patient populations using regulated, oral cannabis-based medicines. As such, research involving more rigorous methodologies is required.

The aim of the current study was to investigate possible impairment to ‘next day’ cognitive and psychomotor function, and simulated driving performance after a single oral dose of a typical cannabis oil by night, in adults with insomnia disorder. This paper describes the secondary outcomes of a larger randomised controlled trial investigating the acute effects of THC/CBD on objective sleep outcomes in insomnia disorder, the results of which will be published separately as those outcomes were beyond the scope of a single manuscript.

## Methods

### Participants

Participants were recruited via self-referral or recommendation from sleep physicians and psychologists, and media advertisements. Inclusion criteria for the study were: (1) between the age of 25 to 65 years; (2) presenting with insomnia disorder, defined clinically as: (a) self-reported difficulty initiating and/or maintaining sleep on >3 nights per week and for >3 months coupled with daytime impairments despite adequate sleep opportunity (5^th^ ed.; DSM-5; American Psychiatric Association 2013); (b) an Insomnia Severity Index (ISI) score ≥15; and (3) in good health as determined via medical history, physical examination, and electrocardiogram. The age range was chosen to limit age-related variability in sleep architecture for better interpretation of EEG changes (Sprecher et al. 2016) in the primary trial. Main exclusion criteria for the study were: (1) reported use of cannabis or illicit drugs in the past three months (abstinence confirmed with a negative urinary drug screen for common drugs of abuse at screening and at the beginning of each study assessment visit); (2) diagnosis of a sleep disorder other than insomnia including advanced or delayed sleep phase syndrome (determined on clinical interview with a sleep specialist and confirmed via in-laboratory diagnostic sleep study); (3) current use of medications that affect the central nervous system (e.g., hypnotics, antidepressants); and (4) pregnant or lactating (assessed with urinary pregnancy tests, as applicable).

### Study design and procedures

This within-participant, double-blind, placebo-controlled, crossover study was conducted from August 2019 to October 2021 at the Woolcock Institute of Medical Research, a specialist outpatient sleep clinic, and followed the Consolidated Standards of Reporting Trials (CONSORT) reporting guideline. The study was approved by Bellberry Human Research Ethics Committee (2018-04-284), and all participants provided written informed consent prior to study procedures. The larger trial was prospectively registered on the Australian New Zealand Clinical Trials Registry (ACTRN12619000714189) in March 2019. The trial protocol was published elsewhere (Suraev et al. 2020).

Participants completed two 24-hour outpatient overnight study assessment visits during which they received 2 mL of oil containing 10 mg THC + 200 mg CBD (‘THC/CBD’; 1:20 ratio of THC to CBD) or matched placebo (2 mL containing no cannabinoids). Each visit was separated by a ≥7-day washout period. The 1:20 THC:CBD ratio has been extensively studied in clinical populations with comorbid insomnia symptoms (Barchel et al. 2019; Hausman-Kedem et al. 2018; Libzon et al. 2018; Tzadok et al. 2016) and is currently available on prescription in Australia (Australian Government; Department of Health and Aged Care 2022). The dose was selected based on prior studies showing that 10 mg oral THC produced discriminable subjective drug effects (e.g., increased “drowsiness”) without altering cognitive and psychomotor performance among infrequent cannabis users (Schlienz et al. 2020; Spindle et al. 2021).

Participants were randomly allocated in a 1:1 ratio to one of two treatment sequences (‘THC/CBD–placebo’ or ‘placebo–THC/CBD’) according to a computer-generated randomization schedule created by an unblinded study investigator (NM) and held in a central location. The amber glass bottles were provided in sequentially numbered boxes, prepared by an independent drug distributor according to the randomization list. Neither the unblinded study investigator nor the drug distributor had any contact with the participants. The trial coordinator (AS) enrolled participants and the study physicians assigned participants to treatment sequence. All participants, trial personnel (including trial coordinator and study physicians), and the outcome assessors were blind to the treatment allocation until statistical analyses for all primary and secondary outcomes were completed.

**Fig. 1:**
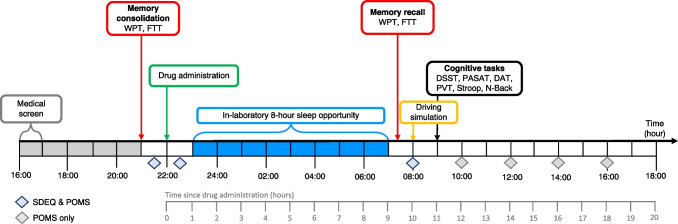
Study procedures and timeline. *DAT* Divided Attention Task, *DSST* Digit Symbol Substitution Task, *FTT* Finger Tapping Task, *POMS* Profile of Mood States, *PSAT* Paced Serial Addition Task; *WPT* Word Pairs Task

At the start of each study assessment visit, participants completed a urine drug screening (DrugCheck NxStep Urine Drug Screen) and pregnancy test (as applicable; Human Chorionic Gonadotrophin Cassette, Alere^TM^) to confirm eligibility. Consumption of caffeinated beverages (e.g., tea, coffee, soft drinks) were not permitted during study assessment visits. Participants were provided standardized meals (evening: 18:30 dinner; next day: ~07:00 breakfast and ~12:45 lunch) and light snacks (e.g., popcorn, fruit). Participants set their preferred bedtime in accordance with a 7-day sleep diary completed prior to their first study assessment visit. This preferred bedtime was adhered to at both study assessment visits. After lights out, participants slept undisturbed in a private bedroom in the outpatient sleep laboratory for a minimum of 8 h before either waking on their own or a sleep technician gradually increased the light in the room before gently waking them. The morning after drug administration, participants completed a range of assessments starting from ~9 h post-drug administration. The trial procedures are summarized in Fig. 1.

### Study drug

The investigational product was a plant-derived oral formulation containing a 1:20 ratio of THC to CBD (i.e., 5 mg/mL THC and 100 mg/mL CBD) suspended in medium-chain triglyceride (MCT) oil and matched placebo; manufactured at a GMP-certified facility (Linnea SA, Lavertezzo, Switzerland). The investigational product was stored at room temperature, as per the manufacturer’s instruction, inside a locked safe on-site at the sleep clinic. The study physician(s) prepared the study drug on the same day of the study assessment visit by drawing 2 mL of active drug or placebo in an amber plastic syringe secured with a tip cap. The active and placebo treatments did not differ in their visual appearance. To improve blinding, participants were instructed to ingest one peppermint lozenge (Fisherman’s Friend Mint; Lofthouse of Fleetwood, England) prior to treatment to mask any possible differences in taste and/or smell. One hour prior to their habitual bedtime, participants were then instructed to slowly press the plunger of the plastic syringe to release the dose under the tongue before immediately swallowing and drinking a small glass of water.

### Outcome measures

#### Cognitive and psychomotor function

A series of cognitive tests were administered within the first 2 h of waking (9-11 h post-drug administration). The following tasks were administered: *Digit Symbol Substitution Task* (attention, working memory, and visuospatial function), *Divided Attention Task* (working memory), *Paced Serial Addition Task* (working memory, attention, and simple arithmetic problem-solving), *Word Pairs Task* (declarative memory), *Finger Tapping Task* (procedural memory), *Stroop Test* (executive function), and *N-Back Task* (working memory and information processing) (full description of each task in Supplement 1).

#### Simulated driving performance

Driving performance was measured 10 h post-treatment using a fixed-base driving simulator (Hyperdrive, Adelaide, Australia) equipped with standard vehicle controls and a custom-built 30-minute scenario that has previously demonstrated sensitivity to the acute effects of THC in healthy volunteers (SCANeR Studio Simulation Engine, v1.6, OKTAL, Paris, France) (Arkell et al. 2019). The driving test incorporated two tasks detailed elsewhere (McCartney et al. 2022a, 2022b): (1) a 7-min ‘car following’ (CF) component during which participants maintained what they considered a ‘safe distance’ between themselves and a lead vehicle accelerating and decelerating (90–110 km/h) at 30 second intervals, and (2) a ~25 minute ‘standard’ drive component along highway (110 km/h signed speed limit) and rural (60–100 km/h signed speed limits) roads. Participants were instructed to follow all road rules and drive in the center of their lane. The outcome measures included (1) standard deviation of lateral position (SDLP) in cm; an index of ‘weaving’, (2) average and standard deviation of headway in m; distance to the lead vehicle in the CF component, and (3) average speed (km/h) and standard deviation of speed; a measure of longitudinal vehicle control. To familiarise themselves with the driving simulator, participants completed a 10-minute practise drive on a separate visit prior to their first study assessment visit.

#### Subjective outcomes

Subjective drug effects were assessed using the Subjective Drug Effect Questionnaire (SDEQ) which uses a 100-mm visual analog scale with the horizontal line anchored with 0 (“not at all”) on the left and 100 (“extremely”) on the right. Participants were asked to rate how “stoned”, “sedated’, “alert”, ‘anxious” and “sleepy’ they felt at baseline, 0.5 h and 10 h post-treatment. Measurements stopped after 10 h because subjective drug effects were not expected to persist beyond this time following a single oral dose of THC.

The 40-item Profile of Mood States (POMS) abbreviated version was used to evaluate how participants felt (“right now”) across seven different mood subscales at seven timepoints: baseline, and 0.5 h, 10 h, 12 h, 14 h, 16 h, and 18 h post-treatment (Grove and Prapavessis 1992). *Total mood disturbance* (TMD) was calculated by summing the negative subscales and subtracting the positive subscales. A constant (i.e., 100) was added to the TMD formula to eliminate negative scores.

### Statistical analysis

Analyses were conducted using SPSS version 26 (IBM Corp., Armonk, NY, USA). Data analysis was initiated on 17 February 2022. All analyses followed the *a priori* defined statistical analysis plan. As this was a pilot study, no formal sample size calculation was performed. Linear mixed models were used to determine differences between treatment and placebo. No interim analyses were planned or undertaken, and there were no stopping guidelines. Outcome measures were analysed in the same order described in the current paper. Fixed factors included *Treatment* (2 levels: THC/CBD and placebo) and *Order* (2 levels: ‘THC/CBD-placebo’ or ‘placebo-THC/CBD’). The fixed factor *Time* was included for outcome measures that were repeated across the visit (3 and 7 levels for DEQ and POMS, respectively), and the *Treatment* × *Time* interaction. *Participant* was included as a random effect in the model. The least-squares means procedure was used in the mixed-model analyses to handle missing data. For the SDEQ and POMS, two-sided pairwise comparisons were used to compare estimated marginal means at *Time* × *Treatment.* Statistical significance was set at less than .05. Figures were created using GraphPad Prism version 9 (GraphPad Inc., San Diego, CA).

Cohen's d_z_ effect sizes were calculated by standardizing the mean difference between THC/CBD and placebo against the standard deviation (SD) of change (Lakens 2013). The 95% confidence intervals (95%CI) were then derived using the Hedges and Olkin approximation adapted for a repeated-measures design (Goulet-Pelletier and Cousineau 2018), as demonstrated previously (McCartney et al. 2022a, 2022b). Data are presented as mean ± SD, unless otherwise stated.

## Results

### Participants

**Fig. 2:**
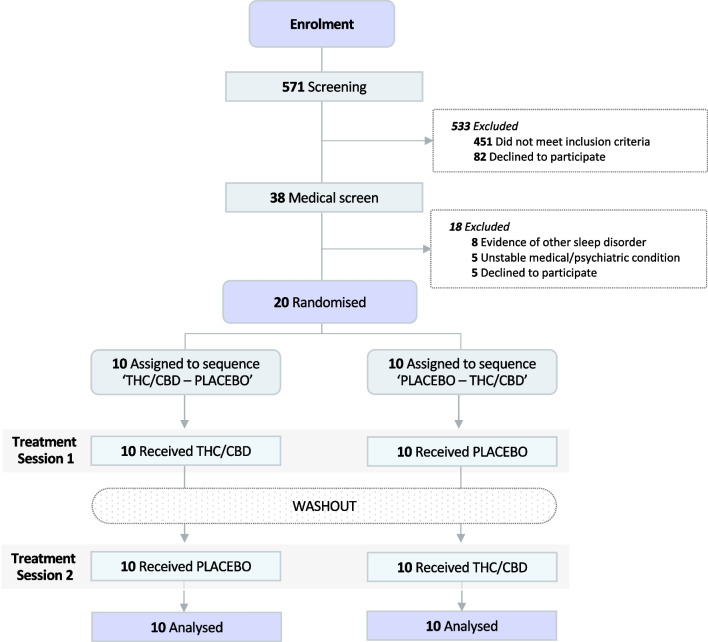
CONSORT Flow Diagram

Twenty participants with insomnia disorder (16 female; mean [SD] age, 46.1 (8.6) years) were recruited and randomised between August 2019 and October 2021 (Table 1 and Fig. 2). All 20 randomised participants completed the trial. The trial was stopped once the predetermined sample size was met. Most participants (75%) were either cannabis-naïve or had <10 lifetime exposures to cannabis while 5 participants (25%) had >10 lifetime exposures. None had used cannabis or cannabinoid products in the last three months; confirmed by a urinary drug screen.

**Table 1:** Participant demographics and characteristics

Variable	Descriptive statistics (*n*=20)
Sex, *n*	
Females	16
Males	4
Age, *y* mean (SD) [range]	46.1 (8.6) [29-62]
BMI, *kg/m*^*2*^	25.1 (3.7)
Participants with at least some tertiary education, *n (%)*	18 (90%)
Participants with current employment, *n (%)*	15 (75%)
Lifetime cannabis exposure, *n* (%)	
Never tried	4 (20%)
10 uses	11 (55%)
>10 uses	5 (25%)

*BMI* Body Mass Index, *SD* standard deviation

### Cognitive and psychomotor function

Table 2 presents outcomes for cognitive and psychomotor function tasks. There was a small reduction in percentage accuracy on the Stoop-Colour test for THC/CBD relative to placebo (mean difference = -1.4% [95%CI -1.1 to -0.1], *p*=0.016, *d* = -0.60). No other significant differences were observed for any of the cognitive performance tasks (all *p*’s>0.10 and Cohen’s *d* effect sizes <0.30).

**Table 2:** Cognitive performance outcomes the next day after evening administration of THC/CBD and placebo

	Placebo; Actual/potential obs	THC/CBD; Actual/potential obs	*p* value	Cohen’s *d* [95% CI]
DSST				
Number correct, *n*	29.3 (10.5); 20/20	29.5 (7.1); 20/20	0.901	-0.03 [-0.46, 0.41]
% Response accuracy	91.8 (8.9); 20/20	90.7 (8.7); 20/20	0.723	-0.08 [-0.52, 0.36]
DAT				
Tracking errors, *pixels*	30.0 (11.0); 20/20	34.2 (25.1); 20/20	0.447	0.17 [-0.27, 0.61]
Response time, *ms*	1288.5 (316.5); 20/20	1213.6 (268.1); 20/20	0.317	-0.23 [-0.68, 0.23]
Number correct, *n*	21.6 (2.3); 20/20	21.9 (2.1); 20/20	0.597	0.13 [-0.32, 0.58]
PSAT				
Number correct, *n*	43.6 (12.2); 20/20	42.0 (16.1); 20/20	0.550	-0.14 [-0.58, 0.31]
Response time, *ms*	1632.1 (126.6); 20/20	1653.0 (156.2); 20/20	0.477	0.16 [-0.28, 0.60]
WPT				
% Retention	90.4 (9.9); 20/20	92.3 (9.9); 20/20	0.377	0.16 [-0.28, 0.60]
FTT				
Pre-training learning	18.2 (1.1); 18/20	18.1 (1.1); 20/20	0.839	-0.11 [-0.55, 0.33]
Post-training learning	21.9 (4.0); 18/20	22.2 (4.0); 20/20	0.267	0.13 [-0.31, 0.57]
Early retest learning	20.0 (7.2); 18/20	20.4 (5.9); 20/20	0.739	0.07 [-0.37, 0.51]
Late retest learning	31.7 (18.4); 18/20	33.4 (17.4); 20/20	0.476	0.15 [-0.29, 0.59]
Overnight early improvement	100.9 (15.8); 18/20	91.4 (19.3); 20/20	0.210	-0.25 [-0.69, 0.19]
Overnight late improvement	152.2 (66.7); 18/20	150.0 (70.1); 20/20	0.695	-0.05 [-0.49, 0.39]
Stroop Test				
% Colour accuracy	**99.2 (1.8); 20/20**	**97.8 (2.3); 20/20**	**0.016**	**-0.60 [-1.08, -0.13]**
Colour RT, *s*	1.3 (0.3); 20/20	1.4 (0.3); 20/20	0.288	0.25 [-0.20, 0.70]
% Word accuracy	99.8 (0.8); 20/20	93.6 (20); 20/20	0.182	-0.31 [-0.76, 0.14]
Word RT, *s*	1.4 (0.2); 20/20	1.4 (0.3); 20/20	0.967	0.01 [-0.43, 0.45]
N-back				
% 1-Back accuracy	87.6 (9.7); 19/20	86.5 (10.9); 20/20	0.472	-0.14 [-0.58, 0.31]
% 2-Back accuracy	89.4 (7.8); 19/20	89.5 (7.8); 20/20	0.849	0.02 [-0.42, 0.46]

Actual/potential obs depicts missing data by giving the amount of observed and recorded data being used by the mixed model compared with the potential number of observations (i.e., complete data). Values are mean (SD) unless otherwise stated. Abbreviations: *DAT* Divided Attention Task, *DSST* Digit Symbol Substitution Task, *FTT* Finger Tapping Task, *PSAT* Paced Serial Addition Task, *RT* reaction time, *WPT* Word Pairs Task. Bold font indicates significant difference *p*<0.05

### Simulated driving performance

Outcome measures for the simulated driving task are presented in Table 3. None of the simulated driving outcome measures were significantly different between THC/CBD and placebo (all *p*’s>0.30 and Cohen’s *d* effect sizes <0.20).

**Table 3:** Measures of next day simulated driving performance

	Placebo; Actual/potential obs	THC/CBD; Actual/potential obs	*p* value	Cohen’s *d* [95% CI]
Car Following Component				
SDLP (cm)	21.6 (4.7); 20/20	22.5 (5.4); 20/20	0.358	0.22 [-0.23, 0.66]
Headway (m)	127.2 (103.6); 20/20	130.5 (80.6); 20/20	0.907	0.03 [-0.41, 0.47]
SD Headway (m)	34.6 (30.7); 20/20	34.8 (28.6); 20/20	0.982	0.01 [-0.43, 0.44]
Standard Component ^a^				
SDLP (cm)	32.2 (4.9); 18/20	33.0 (4.8); 18/20	0.486	0.17 [-0.29, 0.64]
Speed (km/h)	97.4 (3.5); 18/20	97.2 (4.4); 18/20	0.774	-0.07 [-0.53, 0.39]
SD Speed (km/h)	13.1 (2.7); 18/20	13.8 (4.0); 18/20	0.392	0.21 [-0.26, 0.68]

Actual/potential obs depicts missing data by giving the amount of observed and recorded data being used by the mixed model compared with the potential number of observations (i.e., complete data). Values are Mean (SD). Abbreviation: *SDLP* Standard Deviation of Lateral Position, *SD* Standard Deviation. This task was completed ~10 h post-treatment. ^a^Sample size was *n*=18 because two participants failed to complete the “Standard Component” of the simulated driving task on each occasion due to motion sickness

### Subjective outcomes

On the SDEQ, there was a *Treatment x Time* interaction for subjective ratings of ‘Sedated’ with higher ratings for THC/CBD relative to placebo at 10 h post-treatment treatment (mean difference = 8.6 [95% CI -0.12, 0.81], *p*=0.042, *d*=0.35) (Table 4). No other significant *Treatment* × *Time* interactions were observed (all *p*>0.05).

**Table 4:** Subjective drug effects as measured on a visual analog scale (0-100 mm) after evening administration of THC/CBD and placebo

	Placebo; Actual/potential obs	THC/CBD; Actual/potential obs	*p* value	Cohen’s *d* [95% CI]
Stoned				
BL	0.4 (0.8); 19/20	0 (0); 19/20	0.879	^
0.5 h	1.2 (2.7); 19/20	0.2 (0.6); 19/20	0.707	-0.36 [-0.82, 0.12]
10 h	4.5 (12.2); 19/20	7.5 (15); 19/20	0.256	0.14 [-0.21, 0.59]
Sedated				
BL	0.3 (0.8); 19/20	1.7 (5.6); 19/20	0.748	0.23 [-0.22, 0.69]
0.5 h	9.8 (13.8) ; 19/20	2.2 (3.9); 19/20	0.070	-0.63 [-1.12, -0.14]
10 h	**11 (20.3); 19/20**	**19.6 (19); 19/20**	**0.042**	**0.35 [-0.12, 0.81]**
Alert				
BL	51.8 (25.5); 19/20	58 (26.8); 19/20	0.296	0.25 [-0.21, 0.70]
0.5 h	27 (18); 19/20	33.7 (19.7); 19/20	0.267	0.30 [-0.16, 0.76]
10 h	25.8 (19); 19/20	30.1 (21.1); 19/20	0.467	0.18 [-0.28, 0.63]
Anxious				
BL	3.8 (5.5); 19/20	9.2 (15.7); 19/20	0.097	0.37 [-0.10, 0.83]
0.5 h	0.8 (1.8); 19/20	1.8 (3.2); 19/20	0.755	0.27 [-0.18, 0.73]
10 h	4.5 (8.3); 19/20	5.8 (16.4); 19/20	0.689	0.10 [-0.38, 0.52]
Sleepy				
BL	36.7 (27.2); 19/20	39.8 (25.3); 19/20	0.670	0.12 [-0.33, 0.57]
0.5 h	50.3 (25.5); 19/20	44.9 (25.9); 19/20	0.464	-0.23 [-0.69, 0.22]
10 h	50.2 (22.2); 19/20	47.1 (27.5); 19/20	0.667	-0.11 [-0.57, 0.34]

Actual/potential obs depicts missing data by giving the amount of observed and recorded data being used by the mixed model compared with the potential number of observations (i.e., complete data). *BL* baseline. Bold font indicates significant difference *p*<0.05. ^Effect size could not be calculated as the SD of change was zero or very close to zero for both treatments

No significant *Treatment* × *Time* interaction was observed at any timepoint for the TMD score of the POMS.

## Discussion

This randomised controlled trial explored possible ‘next day’ impairment following a single oral dose of an oil containing combined 10 mg THC and 200 mg CBD in adults with insomnia disorder. We found a lack of notable ‘next day’ impairment (>9 h post-treatment) consistent with prior work showing that the impairing effects of oral THC on cognition and driving performance typically resolve within ~8 h (McCartney et al. 2021). These findings confirm and extend on prior work by employing a randomised controlled trial design, a patient population that infrequently use cannabis and who are, on average, older than participants in previous studies (McCartney et al. 2022a, 2022b), and the use of a regulated product containing a higher ratio of CBD to THC which has the potential to potentiate THC blood concentrations and associated impairment (Zamarripa et al. 2023). Overall, we found little evidence to suggest that a single dose of 10 mg oral THC, in conjunction with CBD, impairs ‘next day’ function in adults with insomnia who infrequently use cannabis.

Almost all the cognitive tests conducted, involving attention, working memory, speed of information processing, and other domains, showed no ‘next day’ effects of THC/CBD. The one exception was the *Stoop-Colour Test* in the ‘easy/congruent condition’, where the task requires participants to match the *colour* of the word presented. Here, THC/CBD reduced response accuracy by 1.4% relative to placebo. However, a ceiling effect was evident, with participants demonstrating a very high percentage of accuracy (i.e., >97%) on both treatments suggesting that this effect is not clinically meaningful. Importantly, no significant difference in accuracy was observed on the more difficult ‘hard/incongruent condition’ of the *Stroop-Word Test*, which requires participants to match the *meaning* of the word presented, not the printed colour of the word. For comparison, the morning after alcohol consumption (i.e., the hangover state) produced significantly greater interference on the *Stroop-Word Test,* but not the *Stroop-Colour Test,* relative to the alcohol-free control group (i.e., no hangover state) (Devenney et al. 2019).

There were no impairing effects of THC/CBD given by night on simulated driving performance assessed the following morning at ~10 h post-treatment; coinciding with a time that many people might commute on roads (e.g., driving to work in ‘rush-hour’). This is consistent with our recent meta-regression analysis, which concluded that driving-related skills in occasional cannabis users recover within ~8 h after ingesting 20 mg oral THC (McCartney et al. 2021). Another study also showed no significant difference in SDLP following oral administration of 10 mg THC (dronabinol) relative to placebo in infrequent cannabis users at an even shorter interval of 3.5 h (Schnakenberg Martin et al. 2023). Additionally, other recent studies failed to detect cognitive or driving impairment at 24 h or 48 h following substantial ad-libitum consumption of inhaled cannabis, relative to placebo (Brands et al. 2019) (Matheson et al. 2020). The only notable subjective ‘next day’ effect aligned to treatment was the higher subjective feelings of ‘*Sedated’* with THC/CBD at 10 h post-treatment, however, the effect size was small (*d*=0.3), with no accompanying changes in subjective feelings of ‘*Alert’* or ‘*Sleepy’* (both *p*>0.05). It is also worth noting that, in the broader investigation, this change in subjective sedation did not cause any notable impairment in objective measures of cognitive and psychomotor function or driving performance, many of which require sustained vigilance and alertness for proper execution.

In contrast, commonly prescribed sedative-hypnotics are known to impair next-day function. On-road studies revealed that two days of nocturnal benzodiazepine treatment significantly impaired driving ability the morning after (10-11 h post-treatment) and, in some cases, in the afternoon (>16 h post-treatment) (Brookhuis et al., 1990; O'Hanlon 1984; O'Hanlon and Volkerts 1986; Volkerts et al. 1984; Volkerts et al. 1992). A single night of nocturnal zopiclone (7.5 mg) treatment similarly impaired driving performance in the morning relative to placebo (mean ∆SDLP difference: +3.75 cm) (Iwamoto et al. 2022). Conversely, bedtime use of zaleplon (10-20 mg) did not produce a significant difference in driving performance relative to placebo (mean ∆SDLP difference: +0.7 cm) (Vermeeren et al. 2002). Lemborexant (5 mg/day or 10 mg/day), a dual orexin receptor antagonist approved for insomnia, similarly did not demonstrate clinically significant effects on next-day cognitive function, postural stability, or driving performance the morning after bedtime use across nine clinical trials (Moline et al. 2021). Notably, the magnitude and duration of impairment depends on various factors including dosage, half-life, timing of drug administration, and tolerance. The long-term effects of daily use of sedative-hypnotic medications on next-day impairment requires further investigation.

A strength of this study was the use of a randomised, double-blind, placebo-controlled trial design, reducing the risk of possible confounding factors inherent in observational studies (Meuli and Dick 2018). The use of a regulated cannabis product and validated, objective tests of both cognitive and driving performance in a controlled setting where participants remained under 24 h observation overnight are also major strengths. Diagnostic sleep studies were also used to rule out comorbid sleep disorders that are commonly associated with daytime drowsiness such as sleep apnea (Cruz et al. 2021); a common occurrence in our enrolled cohort (21%; 8/38 screened participants). The study has limitations. First, the relatively small sample size may have limited statistical power to detect subtle effects across outcome measures. Second, the study design was such that the individual contribution of THC and CBD to observed effects could not be assessed. There is emerging evidence that THC and CBD can have pharmacokinetic and pharmacodynamic interactions, although, findings are mixed (Zamarripa et al. 2023; Boggs et al. 2018). Generally, at higher CBD/THC ratios such as those used in the present study, CBD may be more likely to potentiate THC blood concentrations and associated impairment due to a CYP-mediated inhibition of Δ^9^-THC metabolism (Zamarripa et al. 2023). Thus, the presence of CBD in the present study would be expected, if anything, to increase the likelihood of detecting ‘next day’ impairment. There is little likelihood of CBD itself causing any deleterious effects on next day outcomes (McCartney et al. 2022a, 2022b). Finally, the present study only examined a single dose. This precludes any conclusions regarding the effects of repeated dosing with THC, with or without CBD, on daytime function in insomnia disorder, which is more representative of how some people use medicinal cannabis for sleep in the community (De Hoop et al. 2018; Turna et al. 2020). However, it is hypothesised that the chances of detecting ‘next-day’ impairment are less likely with repeated dosing due to the development of at least partial tolerance to the impairing effects of THC (Mason et al. 2021; Ramaekers et al. 2020; Ramaekers et al. 2011).

## Conclusions

The use of cannabis by night as a sleep aid is highly prevalent and there are legitimate concerns that this may lead to impaired daytime (‘next day') function, particularly on safety sensitive tasks such as driving. The results of this study indicate that a single oral dose of 10 mg THC (in combination with 200 mg CBD) does not notably impair ‘next day’ cognitive function or driving performance relative to placebo in adults with insomnia disorder who infrequently use cannabis. Larger studies in patient populations are required to determine the effects of repeated dosing with THC (with or without CBD), and at higher doses of THC, on ‘next day’ function.

### Supplementary information

ESM 1 (DOCX 25.4 KB)

ESM 2 (DOCX 26 kb)

## References

[CR1] American Psychiatric Association (2013) Diagnostic and statistical manual of mental disorders, 5th edn. 10.1176/appi.books.9780890425596

[CR2] Arkell TR, Lintzeris N, Kevin RC, Ramaekers JG, Vandrey R, Irwin C, Haber PS, McGregor IS (2019) Cannabidiol (CBD) content in vaporized cannabis does not prevent tetrahydrocannabinol (THC)-induced impairment of driving and cognition. Psychopharmacology 236(9):2713–2724. 10.1007/s00213-019-05246-8 31044290 10.1007/s00213-019-05246-8 PMC6695367

[CR3] Arkell TR, McCartney D, McGregor IS (2021) Medical cannabis and driving. Aust J Gen Prac 50(6):357–362. 10.31128/AJGP-02-21-5840 10.31128/AJGP-02-21-5840 34059836

[CR4] Australian Government Therapeutic Goods Administration. (2022) Medicinal cannabis products by active ingredients. https://www.tga.gov.au/medicinal-cannabis-products-active-ingredients. Accessed 12 Jan 2024

[CR5] Barchel D, Stolar O, De-Haan T, Ziv-Baran T, Saban N, Fuchs DO, Koren G, Berkovitch M (2019) Oral cannabidiol use in children with autism spectrum disorder to treat related symptoms and co-morbidities. Front Pharmacol 9:1521. 10.3389/fphar.2018.01521 30687090 10.3389/fphar.2018.01521 PMC6333745

[CR6] Boggs DL, Nguyen JD, Morgenson D, Taffe MA, Ranganathan M (2018) Clinical and preclinical evidence for functional interactions of cannabidiol and Δ9-tetrahydrocannabinol. Neuropsychopharmacology 43(1):142–154. 10.1038/npp.2017.209 28875990 10.1038/npp.2017.209 PMC5719112

[CR7] Bosker WM, Kuypers KP, Theunissen EL, Surinx A, Blankespoor RJ, Skopp G, Jeffery WK, Walls HC, van Leeuwen CJ, Ramaekers JG (2012) Medicinal Δ9-tetrahydrocannabinol (dronabinol) impairs on-the-road driving performance of occasional and heavy cannabis users but is not detected in Standard Field Sobriety Tests. Addiction 107(10):1837–1844. 10.1111/j.1360-0443.2012.03928.x 22553980 10.1111/j.1360-0443.2012.03928.x

[CR8] Brands B, Mann RE, Wickens CM, Sproule B, Stoduto G, Sayer GS, Burston J, Pan JF, Matheson J, Stefan C (2019) Acute and residual effects of smoked cannabis: Impact on driving speed and lateral control, heart rate, and self-reported drug effects. Drug Alcohol Depend 205:107641. 10.1016/j.drugalcdep.2019.107641 31678833 10.1016/j.drugalcdep.2019.107641

[CR9] Brookhuis KA, Volkerts ER, O’Hanlon JF (1990) Repeated dose effects of lormetazepam and flurazepam upon driving performance. Eur J Clin Pharm 39:83–87 10.1007/BF02657065 1980464

[CR10] Cole TB, Saitz R (2020) Cannabis and impaired driving. JAMA 324(21):2163–2164. 10.1001/jama.2020.18544 33258875 10.1001/jama.2020.18544

[CR11] De Hoop B, Heerdink ER, Hazekamp A (2018) Medicinal cannabis on prescription in the Netherlands: statistics for 2003–2016. Cannabis Cannabinoid Res 3(1):54–55. 10.1089/can.2017.0059 29588916 10.1089/can.2017.0059 PMC5868329

[CR12] Devenney LE, Coyle KB, Verster JC (2019) Memory and attention during an alcohol hangover. Hum Psychopharmacol Clin Experiment 34(4):e2701. 10.1002/hup.2701 10.1002/hup.2701 PMC6771905 31297901

[CR13] e Cruz MM, Kryger MH, Morin CM, Palombini L, Salles C, Gozal D (2021) Comorbid Insomnia and Sleep Apnea: Mechanisms and implications of an underrecognized and misinterpreted sleep disorder. Sleep Med 84:283–288. 10.1016/j.sleep.2021.05.043 34214960 10.1016/j.sleep.2021.05.043

[CR14] Goulet-Pelletier J-C, Cousineau D (2018) A review of effect sizes and their confidence intervals, Part I: The Cohen’sd family. Quant Meth Psych 14(4):242–265. 10.20982/tqmp.14.4.p242 10.20982/tqmp.14.4.p242

[CR15] Grove JR, Prapavessis H (1992) Preliminary evidence for the reliability and validity of an abbreviated profile of mood states. Int J Sport Psychol 23(2):93–109

[CR16] Hausman-Kedem M, Menascu S, Kramer U (2018) Efficacy of CBD-enriched medical cannabis for treatment of refractory epilepsy in children and adolescents–An observational, longitudinal study. Brain Dev 40(7):544–551. 10.1016/j.braindev.2018.03.013 29674131 10.1016/j.braindev.2018.03.013

[CR17] Iwamoto K, Iwata M, Kambe D, Imadera Y, Tachibana N, Kajiyama Y, Ando M, Ozaki N (2022) Residual effects of zopiclone on driving performance using a standardized driving simulator among healthy volunteers. Psychopharmacology 239(3):841–850. 10.1007/s00213-022-06075-y 35106620 10.1007/s00213-022-06075-y

[CR18] Lakens D (2013) Calculating and reporting effect sizes to facilitate cumulative science: a practical primer for t-tests and ANOVAs. Front Psychol 4:863. 10.3389/fpsyg.2013.00863 24324449 10.3389/fpsyg.2013.00863 PMC3840331

[CR19] Libzon S, Schleider LB-L, Saban N, Levit L, Tamari Y, Linder I, Lerman-Sagie T, Blumkin L (2018) Medical cannabis for pediatric moderate to severe complex motor disorders. J Child Neurol 33(9):565–571. 10.1177/08830738187730 29766748 10.1177/08830738187730

[CR20] Mason NL, Theunissen EL, Hutten NR, Tse DH, Toennes SW, Jansen JF, Stiers P, Ramaekers JG (2021) Reduced responsiveness of the reward system is associated with tolerance to cannabis impairment in chronic users. Addict Biol 26(1):e12870. 10.1111/adb.12870 31865628 10.1111/adb.12870 PMC7757162

[CR21] Matheson J, Mann RE, Sproule B, Huestis MA, Wickens CM, Stoduto G, George TP, Rehm J, Le Foll B, Brands B (2020) Acute and residual mood and cognitive performance of young adults following smoked cannabis. Pharmacol Biochem Behav 194:172937 32360692 10.1016/j.pbb.2020.172937

[CR22] McCartney D, Arkell TR, Irwin C, McGregor IS (2021) Determining the magnitude and duration of acute Δ9-tetrahydrocannabinol (Δ9-THC)-induced driving and cognitive impairment: a systematic and meta-analytic review. Neurosci Biobehav Rev 126:175–193. 10.1016/j.pbb.2020.172937 33497784 10.1016/j.pbb.2020.172937

[CR23] McCartney D, Suraev A, McGregor IS (2022a) The “next day” effects of cannabis use: a systematic review. Cannabis Cannabinoid Res 8(1):92–114. 10.1089/can.2022.0185 36475998 10.1089/can.2022.0185 PMC9940812

[CR24] McCartney D, Suraev AS, Doohan PT, Irwin C, Kevin RC, Grunstein RR, Hoyos CM, McGregor IS (2022b) Effects of cannabidiol on simulated driving and cognitive performance: A dose-ranging randomised controlled trial. J Psychopharmacol 36(12). 10.1177/02698811221095356 10.1177/02698811221095356 PMC9716488 35637624

[CR25] Meuli L, Dick F (2018) Understanding confounding in observational studies. Eur J Vasc Endovasc Surg 55(5):737. 10.1016/j.ejvs.2018.02.028 29526654 10.1016/j.ejvs.2018.02.028

[CR26] Moline M, Zammit G, Yardley J, Pinner K, Kumar D, Perdomo C, Cheng JY (2021) Lack of residual morning effects of lemborexant treatment for insomnia: summary of findings across 9 clinical trials. Postgrad Med 133(1):71–81. 10.1080/00325481.2020.1823724 33119423 10.1080/00325481.2020.1823724

[CR27] O'Hanlon J (1984) Driving performance under the influence of drugs: rationale for, and application of, a new test. Br J Clin Pharmacol 18(S1):121S–129S. 10.1111/j.1365-2125.1984.tb02590.x 6525328 10.1111/j.1365-2125.1984.tb02590.x PMC1463355

[CR28] O'Hanlon JF, Volkerts E (1986) Hypnotics and actual driving performance. Acta Psychiatr Scand 74(S332):95–104. 10.1111/j.1600-0447.1986.tb08985.x 10.1111/j.1600-0447.1986.tb08985.x 3554901

[CR29] Preuss UW, Hoch E, Wong J (2023) Cannabis, cognitive impairment and car crash risk. In: Cannabis Use, Neurobiology, Psychology, and Treatment. Elsevier Inc., pp 113–124. 10.1016/B978-0-323-89862-1.00027-1

[CR30] Ramaekers J, Mason N, Theunissen E (2020) Blunted highs: pharmacodynamic and behavioral models of cannabis tolerance. Eur Neuropsychopharmacol 36:191–205. 10.1016/j.euroneuro.2020.01.006 32014378 10.1016/j.euroneuro.2020.01.006

[CR31] Ramaekers JG (2018) Driving under the influence of cannabis: an increasing public health concern. JAMA 319(14):1433–1434. 10.1001/jama.2018.1334 29582068 10.1001/jama.2018.1334

[CR32] Ramaekers JG, Theunissen EL, De Brouwer M, Toennes SW, Moeller MR, Kauert G (2011) Tolerance and cross-tolerance to neurocognitive effects of THC and alcohol in heavy cannabis users. Psychopharmacology 214:391–401. 10.1007/s00213-010-2042-1 21049267 10.1007/s00213-010-2042-1 PMC3045517

[CR33] Rogeberg O (2019) A meta-analysis of the crash risk of cannabis-positive drivers in culpability studies—avoiding interpretational bias. Accid Anal Prev 123:69–78. 10.1016/j.aap.2018.11.011 30468948 10.1016/j.aap.2018.11.011

[CR34] Rogeberg O, Elvik R (2016) The effects of cannabis intoxication on motor vehicle collision revisited and revised. Addiction 111(8):1348–1359. 10.1111/add.13347 26878835 10.1111/add.13347

[CR35] Schlienz NJ, Spindle TR, Cone EJ, Herrmann ES, Bigelow GE, Mitchell JM, Flegel R, LoDico C, Vandrey R (2020) Pharmacodynamic dose effects of oral cannabis ingestion in healthy adults who infrequently use cannabis. Drug Alcohol Depend 211:107969. 10.1016/j.drugalcdep.2020.107969 32298998 10.1016/j.drugalcdep.2020.107969 PMC8221366

[CR36] Schnakenberg Martin AM, Flynn LT, Sefik E, Luddy C, Cortes-Briones J, Skosnik PD, Pittman B, Ranganathan M, D’Souza DC (2023) Preliminary study of the interactive effects of THC and ethanol on self-reported ability and simulated driving, subjective effects, and cardiovascular responses. Psychopharmacology 240(6):1235–1246. 10.1007/s00213-023-06356-0 37045988 10.1007/s00213-023-06356-0

[CR37] Spindle TR, Martin EL, Grabenauer M, Woodward T, Milburn MA, Vandrey R (2021) Assessment of cognitive and psychomotor impairment, subjective effects, and blood THC concentrations following acute administration of oral and vaporized cannabis. J Psychopharmacol 35(7):786–803. 10.1177/02698811211021583 34049452 10.1177/02698811211021583 PMC9361180

[CR38] Sprecher KE, Riedner BA, Smith RF, Tononi G, Davidson RJ, Benca RM (2016) High resolution topography of age-related changes in non-rapid eye movement sleep electroencephalography. PLoS One 11(2):e0149770. 10.1371/journal.pone.0149770 26901503 10.1371/journal.pone.0149770 PMC4764685

[CR39] Suraev A, Grunstein RR, Marshall NS, D'Rozario AL, Gordon CJ, Bartlett DJ, Wong K, Yee BJ, Vandrey R, Irwin C (2020) Cannabidiol (CBD) and Δ9-tetrahydrocannabinol (THC) for chronic insomnia disorder (‘CANSLEEP’trial): protocol for a randomised, placebo-controlled, double-blinded, proof-of-concept trial. BMJ Open 10(5):e034421. 10.1136/bmjopen-2019-034421 32430450 10.1136/bmjopen-2019-034421 PMC7239553

[CR40] Turna J, Balodis I, Munn C, Van Ameringen M, Busse J, MacKillop J (2020) Overlapping patterns of recreational and medical cannabis use in a large community sample of cannabis users. Compr Psychiatry 102:152188. 10.1016/j.comppsych.2020.152188 32653594 10.1016/j.comppsych.2020.152188

[CR41] Tzadok M, Uliel-Siboni S, Linder I, Kramer U, Epstein O, Menascu S, Nissenkorn A, Yosef OB, Hyman E, Granot D (2016) CBD-enriched medical cannabis for intractable pediatric epilepsy: the current Israeli experience. Seizure 35:41–44. 10.1016/j.seizure.2016.01.004 26800377 10.1016/j.seizure.2016.01.004

[CR42] Vermeeren A, Riedel WJ, van Boxtel MP, Darwish M, Paty I, Patat A (2002) Differential residual effects of zaleplon and zopiclone on actual driving: a comparison with a low dose of alcohol. Sleep 25(2):224–231. 10.1093/sleep/25.2.224 11905433 10.1093/sleep/25.2.224

[CR43] Volkerts E, Louwerens J, Gloerich A, Brookhuis K, O'hanlon, J. (1984) Zopiclone's residual effect upon actual driving performance versus those of nitrazepam and flunitrazepam. Clin Neuropharmacol 7:S337 10.1097/00002826-198406001-00306

[CR44] Volkerts E, Van Laar M, Van Willigenburg A, Plomp T, Maes R (1992) A comparative study of on-the-road and simulated driving performance after nocturnal treatment with lormetazepam 1 mg and oxazepam 50 mg. Hum Psychopharmacol Clin 7(5):297–309. 10.1002/hup.470070502 10.1002/hup.470070502

[CR45] Zamarripa CA, Spindle TR, Surujunarain R, Weerts EM, Bansal S, Unadkat JD, Paine MF, Vandrey R (2023) Assessment of orally administered Δ9-tetrahydrocannabinol when coadministered with cannabidiol on Δ9-tetrahydrocannabinol pharmacokinetics and pharmacodynamics in healthy adults: A randomized clinical trial. JAMA Netw 6(2):e2254752. 10.1001/jamanetworkopen.2022.54752 10.1001/jamanetworkopen.2022.54752 PMC9926328 36780161

