# Supplementary material for: Evaluating possible ‘next day’ impairment in insomnia patients administered an oral medicinal cannabis product by night: a pilot randomized controlled trial
Source: Psychopharmacology (Berl). 2024 May 17;241(9):1815–25. doi: 10.1007/s00213-024-06595-9 (PMC11339085; doi:10.1007/s00213-024-06595-9)
Supplement: Supplementary file 1 — (DOCX 25.4 KB) [file 213_2024_6595_MOESM1_ESM.docx]

**SUPPLEMENTARY MATERIAL**

**Evaluating possible ‘next day’ impairment in insomnia patients administered an oral medicinal cannabis product by night: a pilot randomized controlled trial**

Anastasia Suraev PhD^1,2,3,4^, Danielle McCartney PhD^2,3,4^, Nathaniel S. Marshall PhD^1,5^, Christopher Irwin PhD^9,10^, Ryan Vandrey PhD^11^, Ronald R. Grunstein MBBS, MD, PhD^1,7,8^, Angela L D’Rozario PhD^1,6^, Christopher Gordon PhD^1,5^, Delwyn Bartlett PhD^1^, *Camilla M Hoyos PhD^1,5^, *Iain S McGregor PhD^2,3,4^

**These authors have contributed equally to this work*

^1^ Centre for Sleep and Chronobiology, Woolcock Institute of Medical Research, Sydney, Australia

^2^ University of Sydney, Lambert Initiative for Cannabinoid Therapeutics, Sydney, Australia

^3^ University of Sydney, Faculty of Science, School of Psychology, Sydney, Australia

^4^ University of Sydney, Brain and Mind Centre, Sydney, Australia

^5^ Macquarie University, Faculty of Medicine, Health and Human Sciences, Department of Health Science, Sydney, Australia

^6^ Macquarie University, Faculty of Medicine, Health and Human Sciences, School of Psychological Sciences, Sydney, Australia
^7^ University of Sydney, Faculty of Medicine and Health, Sydney, Australia

^8^ Royal Prince Alfred Hospital, Department of Respiratory and Sleep Medicine, Sydney, Australia

^9^ Griffith University, School of Health Sciences and Social Work, Gold Coast, Australia

^10^ Menzies Health Institute Queensland, Gold Coast, Australia

^11^ Department of Psychiatry and Behavioral Sciences, Johns Hopkins University School of Medicine, Baltimore, Maryland, USA

**Measures of Cognitive and Psychomotor Function**

*Digit Symbol Substitution Task*

The Digit Symbol Substitution Task measures a range of cognitive skills including speed, attention, working memory, and visuospatial function^1^ and has demonstrated sensitivity to the impairing effects of THC.^2^ Participants were presented with a series of geometric patterns labeled from 1 to 9, each consisting of an array of filled and blank squares in a 3 $\times$3 grid. When a number appeared in the middle of the computer screen, participants were instructed to replicate the pattern corresponding to that array using the numeric keypad of a computer keyboard. Participants had 90 seconds to replicate as many patterns as possible. The outcome measures included the number of correct patterns and accuracy (number of correct patterns/number of patterns attempted).

*Divided Attention Task*

The Divided Attention Task assesses working memory and ability to allocate attention to different aspects of a task.^3^ Participants were required to track a horizontally moving stimulus on the computer screen using their mouse while simultaneously responding to peripheral visual stimuli by clicking the left mouse button whenever a number in any corner of the screen matched a target number presented at the bottom of the screen. Outcome measures included the mean distance of the cursor from the target in pixels (tracking error), proportion of target numbers correctly identified (out of 24), and average response time.

*Paced Serial Addition Task*

The Paced Serial Addition Task measures working memory, attention, and simple arithmetic problem-solving.^4^ Participants observed single digits appear on the computer screen and were instructed to summate each new digit with the preceding one. Participants responded by clicking on the correct answer from a list of numbers (1–10) presented on the screen. The outcome measures included average response time on correct trials and the total number of correct trials (out of 90).

*Word Pairs Task*

The Word Pairs Task measures sleep-dependent declarative memory (procedural memory task) consolidation in adults. The encoding phase was administered 1 hour prior to drug administration at approximately 21:00 on the night of each treatment session. Participants were presented with 32 pairs, one at a time for 5 seconds each, and asked to memorise the pairs. Easy (i.e., semantically related, e.g., fork-knife) and difficult (semantically unrelated, e.g., syrup-feet) word pairs were randomly interspersed, and the order of presentation was randomised for each participant at the first treatment session. The recall (re-test) and recognition phase were administered the following morning at 9.5 h post-drug administration. The outcome measure was the percentage of word pairs correctly recalled in the morning re-test.

*Finger Tapping Task*

The Finger Tapping Task is a psychomotor sequence learning task that assesses procedural memory. Participants are asked to tap a 5-digit sequence (for example 4-1-3-2-4) with their non-dominant hand as rapidly and accurately as possible using the numeric key-buttons of a computer keyboard.^5^ To reduce working memory load, the numeric sequence is displayed at the centre of the screen throughout the task. During a training session completed at 0.5 h prior to drug administration, participants completed 12 blocks, each consisting of a 30 second task followed by 30 second rest. The next morning (at approximately 9.5 h post-drug administration), participants completed a recall session composed of 6 blocks, each consisting of a 30 second task followed by 30 second rest. The outcome measures included: (a) pre-training learning (number of correct sequences averaged across the first three trials prior to sleep); (b) post-training learning (number of correct sequences averaged across the last three trials prior to sleep); (c) early retest learning (number of correct sequences averaged across the last three trials following sleep); (d) late retest learning (number of correct sequences averaged across the last three trials following sleep); (e) overnight early improvement (the percentage overnight improvement in motor skill defined as the early retest learning score/post-training learning score x100), also termed offline memory consolidation; (f) overnight late improvement (the percentage overnight improvement in motor skill defined as the late retest learning score/post-training learning score x100), as previously defined.^6^

*Stroop Test*

The Stroop test assesses the inhibition of dominant responses and reflects the “higher-order” executive functions.^7^ It assesses reaction time to colours, *Stroop-Colour* (easy/congruent condition), and words, *Stroop-Word* ‘hard/incongruent condition’, displayed and cognitive interference due to presentation of simultaneous conflicting information. Words (red, green, or blue) and three different coloured squares (red, green, or blue) were displayed on the computer screen. Participants were required to click on the coloured square that matched either the colour (*Stroop-Colour*) or the meaning (*Stroop-Word*) of the word presented. Each part of the test was 45 seconds in duration and involved multiple trials. The outcome measures included the percentage of correct responses and the average response latency.

*N-Back Task*

The N-Back assesses working memory, encompassing short-term memory storage and information processing.^8^ In this study, the 1-Back and 2-Back were used. Participants were asked to compare the position of a letter displayed on the computer screen to the position of the letter presented one or two trials previously. For example, for 2-Back, the position of the 3^rd^ letter is compared to the position of the 1^st^ letter and the position of the 4^th^ letter to the 2^nd^ letter, and so on. If the position of the letters matched, the participant pressed “M” on the keyboard for “Match” as quickly as possible. If the position of the letters did not match, the participant pressed “N” for “No Match” as quickly as possible. Each N-back task was 4 min in duration and consisted of 50 trials, with a stimulus presented every 4.5 seconds. Percentage accuracy was calculated for both tasks.

**REFERENCES**

1. McLeod DR, Griffiths RR, Bigelow GE, et al. An automated version of the digit symbol substitution test (DSST). *Behav Res Metn* 1982;14(5):463-66.

2. Vandrey R, Herrmann ES, Mitchell JM, et al. Pharmacokinetic profile of oral cannabis in humans: blood and oral fluid disposition and relation to pharmacodynamic outcomes. *J Anal Toxicol* 2017;41(2):83-99.

3. Kleykamp BA, Griffiths RR, Mintzer MZ. Dose effects of triazolam and alcohol on cognitive performance in healthy volunteers. *Exp Clin Psychopharmacol* 2010;18(1):1.

4. Herrmann ES, Cone EJ, Mitchell JM, et al. Non-smoker exposure to secondhand cannabis smoke II: effect of room ventilation on the physiological, subjective, and behavioral/cognitive effects. *Drug Alcohol Depend* 2015;151:194-202.

5. Walker MP, Brakefield T, Seidman J, et al. Sleep and the time course of motor skill learning. *Learn Mem* 2003;10(4):275-84.

6. Terpening Z, Naismith S, Melehan K, et al. The contribution of nocturnal sleep to the consolidation of motor skill learning in healthy ageing and P arkinson's disease. *J Sleep Res* 2013;22(4):398-405.

7. Lezak MD, Howieson DB, Loring DW, et al. Neuropsychological assessment: Oxford University Press, USA 2004.

8. D'Rozario AL, Field CJ, Hoyos CM, et al. Impaired neurobehavioural performance in untreated obstructive sleep apnea patients using a novel standardised test battery. *Front Surg* 2018;5:35.
