# Supplementary material for: Evaluating possible ‘next day’ impairment in insomnia patients administered an oral medicinal cannabis product by night: a pilot randomized controlled trial
Source: Psychopharmacology (Berl). 2024 May 17;241(9):1815–25. doi: 10.1007/s00213-024-06595-9 (PMC11339085; doi:10.1007/s00213-024-06595-9)
Supplement: Supplementary file 2 — (DOCX 26 kb) [file 213_2024_6595_MOESM2_ESM.docx]

**Statistical Analysis Plan**

# Outcome Measures (ACTRN12619000714189)

| **Outcome Measure** | **Timepoint** |
| --- | --- |
| **Primary Outcome Measures** | |
| 1. Change in total sleep time (TST) measured in minutes from in-laboratory overnight PSG | Assessed continuously during both overnight sleep studies (comparison between THC/CBD versus placebo) |
| 1. Change in wake after sleep onset (WASO) measured in minutes from in-laboratory overnight PSG |  |
| **Secondary Outcome Measures** | |
| 1. Average number and duration of sleep and wake bouts from in-laboratory overnight PSG. | This is a composite secondary outcome.  Assessed continuously during both overnight sleep studies (comparison between THC/CBD versus placebo) |
| 1. Global EEG power during NREM and REM sleep (256-channel high-density EEG during overnight PSG) |  |
| 1. Regional EEG power during NREM and REM sleep (256-channel high-density EEG during overnight PSG) |  |
| 1. Sleep spindle and slow oscillation events in NREM sleep from in-laboratory overnight PSG. |  |
| 1. Sleep micro- and macroarchitecture metrics (NREM sleep, slow wave sleep, REM sleep, arousal index, and stage shifts) from standard overnight PSG. |  |
| 1. Subjective ratings of changes in sleep-wake behaviour on the Leeds Sleep Evaluation Questionnaire (LSEQ) | Assessed within 1 hour after waking at both overnight sleep studies (comparison between THC/CBD versus placebo) |
| 1. Subjective ratings of tiredness on the Daytime Insomnia Symptom Response Scale (DISRS) |  |
| 1. Resting wake EEG power before and after sleep using high-density EEG recordings during the Karolinska Drowsiness Test (KDT) in conjunction with the Karolinska Sleepiness Scale (KSS). | Assessed 30 minutes prior to sleep and 30 minutes after waking at both overnight sleep studies (comparison between THC/CBD versus placebo) |
| 1. Mean latency to sleep on the Maintenance of Wakefulness Test (MWT) | Assessed at 10am, 12pm, 2pm, and 4pm after waking at both overnight sleep studies (comparison between THC/CBD versus placebo) |
| 1. Declarative memory performance on the Word Pairs Task (WPT) | Assessed within 2 hours after waking at both overnight sleep studies (comparison between THC/CBD versus placebo) |
| 1. Procedural memory performance on the Finger Tapping Task (FTT) |  |
| 1. Simulated Driving Performance   This is comprised of the following outcome measures: SD of lateral position (SDLP) (measure of lane weaving), Mean speed and SD of speed, Number of lane crossings | Assessed within ~1 hour after waking at both overnight sleep studies (comparison between THC/CBD versus placebo) |
| 1. Next-day cognitive function assessed via computerized tasks:   The Stroop Task (function)  Psychomotor Vigilance Task (sustained attention)  1-and 2-N-back Test (working memory)  Digit Symbol Substitution Task (speed of information processing)  Divided Attention Task (working memory and attention)  Continuous Performance Task Brief (sustained attention)  Paced Auditory Serial Addition Task (information processing/attention) | Assessed within 2 hours after waking at both overnight sleep studies (comparison between THC/CBD versus placebo) |
| 1. Plasma cannabinoid concentrations, including includes measures of THC, THC-COOH, 11-OH-THC, CBD, and endocannabinoids including 2-AG and anandamide. | Blood collected within 1 hour of waking at both overnight sleep studies (comparison between THC/CBD versus placebo) |
| 1. Adverse Event Profile & Treatment Order Guess | Assessed before discharge at both overnight sleep studies (comparison between THC/CBD versus placebo) |
| Exploratory Outcome Measures | |
| 1. Salivary drug tests (Quantisal, Drager and Securetec) | Assessed at four timepoints: baseline, T1 (30 min after drug administration), T2 (10 h after drug administration) and T3 (18 h after drug administration). |
| 1. Mood assessed via the Profile of Mood States (POMS) | Assessed at baseline, and at 0.5 h, 9 h, 12 h, 14 h, 16 h, and 18 h after drug administration. |
| 1. Subjective Drug Effects Questionnaire (SDEQ) | Assessed at baseline, and at 0.5 h and 10 h after drug administration. |

# Co-primary Outcomes

A linear mixed model will be performed in SPSS for the co-primary outcomes, total sleep time (TST) and wake after sleep onset (WASO).

Fixed effects of ***Treatment*** (2 levels: THC/CBD and placebo) and ***Order*** (2 levels: ‘THC/CBD-placebo’ or ‘placebo-THC/CBD’) will be included. ***Participant*** will be a random effect included in the model. The least-squares means procedure will be used in the mixed-model analyses to handle missing data.

SPSS syntax (e.g., TST in minutes):

MIXED Total_Sleep_Time_min BY Participant Treatment Order

/CRITERIA=DFMETHOD(SATTERTHWAITE) CIN(95) MXITER(100) MXSTEP(10) SCORING(1) SINGULAR(0.000000000001) HCONVERGE(0, ABSOLUTE) LCONVERGE(0, ABSOLUTE) PCONVERGE(0.000001, ABSOLUTE)

/FIXED=Treatment Order | SSTYPE(3)

/METHOD=REML

/PRINT=DESCRIPTIVES SOLUTION TESTCOV

/RANDOM=INTERCEPT Participant | COVTYPE(VC)

/EMMEANS=TABLES(Treatment).

# Secondary and Exploratory Outcomes

Linear mixed-model analyses will be used to determine differences between treatments.

Fixed effects of ***Treatment*** (2 levels: THC/CBD and placebo), ***Order*** (2 levels: ‘THC/CBD-placebo’ or ‘placebo-THC/CBD’), ***Time*** (3 and 7 levels for DEQ and POMS, respectively) and ***Treatment*** $\boldsymbol{\times}$ ***Time*** (for DEQ and POMS only) will be included. ***Participant*** (ID 1-20) will be a random effect.

Only two outcome measures, DEQ and POMS, will be repeated across the session. For the DEQ and POMS, two-sided pairwise comparisons will be used to compare estimated marginal means at *Time* $\times$ *Treatment.* The least-squares means procedure will be used in the mixed-model analyses to handle missing data.

SPSS syntax (e.g., SDEQ):

MIXED SDEQ_Sedated BY Participant Treatment Order Time

/CRITERIA=DFMETHOD(SATTERTHWAITE) CIN(95) MXITER(100) MXSTEP(10) SCORING(1) SINGULAR(0.000000000001) HCONVERGE(0, ABSOLUTE) LCONVERGE(0, ABSOLUTE) PCONVERGE(0.000001, ABSOLUTE)

/FIXED=Treatment Order Time Treatment*Time| SSTYPE(3)

/METHOD=REML

/PRINT=DESCRIPTIVES SOLUTION TESTCOV

/RANDOM=INTERCEPT Participant | COVTYPE(VC)

/EMMEANS=TABLES(OVERALL)

/EMMEANS=TABLES(Treatment*Time) COMPARE(Treatment) ADJ(LSD).
